# Management of Root Perforations Using MTA with or without Er:YAG Laser Irradiation: An In Vitro Study

**DOI:** 10.1155/2012/628375

**Published:** 2012-07-19

**Authors:** M. Tielemans, I. Saloukas, D. Heysselaer, Ph. Compere, C. Nyssen-Behets, S. Nammour

**Affiliations:** ^1^Department of Dental Sciences, Faculty of Medicine, University of Liège, 4000 Liège, Belgium; ^2^Unit of Ultrastructural Morphology, Laboratory of Evolutive and Functional Morphology, University of Liège, 4000 Liège, Belgium; ^3^L'Unité de Morphologie Expérimrntale, Université Catholique de Louvain, 1200 Bruxelles, Belgium

## Abstract

The aim of this in vitro study is to compare the microleakage of a root perforation sealed with MTA (mineral trioxide aggregate) (group M) to that sealed with MTA following Er:YAG laser irradiation (group ML). Forty-two recently extracted human monoroot teeth were used. Two cavities were prepared on each root surface. Randomly, on each root, the exposed dentine of one cavity was irradiated prior to MTA filling using an Er:YAG laser with the following settings: 200 mJ/pulses under an air water spray, 10 Hz, pulse duration of 50 **μ**sec, and 0.7 mm beam diameter. All cavities were then sealed with MTA. submitted to thermocycling and immersed in 2% methylene blue dye solution for 12 h. The penetration of methylene blue in the microleakage of cavity was observed and recorded. The mean value dye penetration in cavities sealed with MTA following Er:YAG laser irradiation (23.91 ± 14.63%) was lower than that of unlased cavities sealed only with MTA (25.17 ± 17.53%). No significant difference was noted. The use of an Er:YAG laser beam for dentinal conditioning prior to MTA filling of perforated roots did not decrease significantly the microleakage of MTA sealing when compared to the conventional use of MTA filling.

## 1. Introduction

Root perforations connect root canal spaces with periodontal tissues. The connection may occur as a result of iatrogenic causes during root canal treatment (at the level of the floor of the cameral cavity or at different levels of the root) or during prosthetic treatment for postcanal penetration. It may be also inducted by external root resorption or by the caries process. Prognoses for perforated roots depend on the time lapsed before the perforation is sealed, the localization and size of the perforation, and the quality of the sealing material used.

Mineral trioxide aggregate (MTA) has been used in a variety of surgical and nonsurgical endodontic applications [1]. Several studies have demonstrated that MTA offers good-quality sealing of dentine [2–7]. Unlike other commonly used materials such as glass ionomer and reinforced zinc oxide-eugenol cement (Super-EBA; Harry J; Bosworth Company, Skokie, IL, USA), MTA is a biocompatible material and allows bone formation [8–11]. MTA is commonly accepted as the best choice for root perforation treatment.

On the other hand, Raldi et al. [12] demonstrated that the surface of dentin irradiated by an Er:YAG laser has better cell adhesion than MTA surfaces and unlased dentinal surfaces. Furthermore, Baraba et al. [13] showed that an Er:YAG laser, used under specific irradiation conditions, is more efficient than mechanical drills for enamel and dentin ablation. Delmé et al. [14] demonstrated that the use of acid etching is mandatory to obtain good adhesion and retention with resin composites.

The purpose of this study was to evaluate the capacity of the Er:YAG laser to improve the quality of MTA sealing and to compare the microleakage of roots sealed using MTA versus those sealed by MTA assisted by an Er:YAG laser.

## 2. Materials and Methods

### 2.1. Root Cavities Preparation

**Figure 1:**
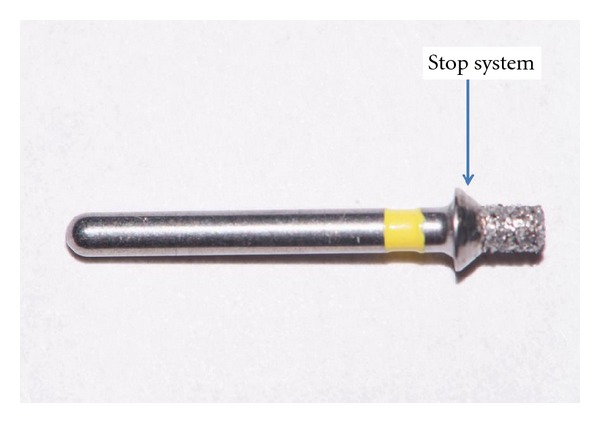
View of the diamond bur used for cavity preparations. Arrow shows the stop system.

Forty-two recently extracted human mono-roots were used (mainly canines and second premolars), stored in distilled water at 4°C. Two cavities were prepared on the opposite sides of the cervical part of each root, using a standard diamond bur of 1.7 mm diameter and 2 mm depth with a stopper (Meissinger 828G017, Hager-Meisinger Gmbh, Neuss Germany) (Figure 1). The bottom of the cavities did not have any connection with root canals.

### 2.2. Laser Irradiation Parameters

The laser apparatus was an Er:YAG laser (wavelength: 2940 nm; Fidelis Plus II, Fotona, Slovania). Laser settings were 200 mJ, 10 Hz, SSP mode (50 *μ*sec), energy density per pulse: 44,1 J/cm^2^, water spray: 10 mL/min, air: 20 mL/min, noncontact hand piece (model R 02-C), beam diameter of 0.7 mm, and distance to target: 7–9 mm.

On each root, one of the cavities was randomly selected. Only the surface of the cavity was irradiated superficially in one passage. A total number of 39 teeth were used for sealing measurements, and three teeth were used for SEM views (group ML).

### 2.3. Preparation of MTA

One commercial brand of gray MTA (Proroot; Dentsply Maillefer, Ballaigues, Switzerland) was used. The cement was prepared according to the manufacturer recommendations. MTA was placed in each cavity. Light pressure was applied to the MTA using wet cotton pellets. The surface was burnished with a B-3 condenser/ball burnisher (Analytic, Synbron Endo, Orange, USA) in order to improve the marginal sealing. The roots were stored in moist conditions at 37°C for one week.

Before MTA sealing of the cavities, the dentine of the drilled and unlased cavities was left without any complementary treatment (group M). The smear layer was left on the dentinal surface.

### 2.4. Thermocycling

One week after MTA sealing of the cavities and before thermocycling, the root surfaces were polished by means of sof-lex Pop-on discs (medium, 3 M, ESPE, USA) under cold water, with the aim of avoiding an eventual excess of MTA overhanging the border of the cavities. Next, the roots were thermocycled for 1000 cycles, for 24 h, from 5°C to 55°C (Willytec Thermocycler V 2.9, Westerham, Germany).

### 2.5. Microleakage Measurements

Two coats of an acid-resistant marine varnish (Alkydurethan varnish, Trimetal, Belgium) were applied, leaving a 0.5 mm border around the edge of each cavity in order to protect the rest of the tooth from the dye solution. The roots were immersed in 2% methylene blue solution for 12 h. Great care was taken to keep the apices of the roots out of the dye solution (the coronal parts of the teeth were held in wax to prevent the root apices from being immersed). The teeth were rinsed and brushed (Oral B, Braun).

Each root was sliced longitudinally in order to allow observation of both cavities. An average of three slices were collected from each root. All specimens were examined blindly by three examiners. Penetration of the dye was measured in millimeters using Visilog 5.3 analysis software (Noesis vision, St Laurent, PQ, Canada). For each cavity, the deepest penetration of the methylene blue dye was recorded (*D* value) after agreement of the examiners. The measurements were calibrated by reference to the total length of the diamond bur (2 mm). The percentage of dye penetration was calculated by dividing the deepest penetration of the dye by the total depth of the cavity (2 mm) (*D*/2 mm × 100 = % of dye penetration). Percentages of dye penetration in all cavities were used for statistical analysis (*t*-Student). A normality test, using the Kolmogorov and Smirnov method, was performed and followed by a test for the qualitative paired data.

The comparison of means and SD of microleakage was expressed in percentage of dye penetration.

### 2.6. SEM Analysis

Three more monoroots were used for SEM analysis (Hitachi S-4700-II FESEM, Tokyo, Japan). The aspect of the drilled dentine without any complementary treatment was compared to the aspect of the drilled and Er:YAG-conditioned dentine.

## 3. Results

**Figure 2:**
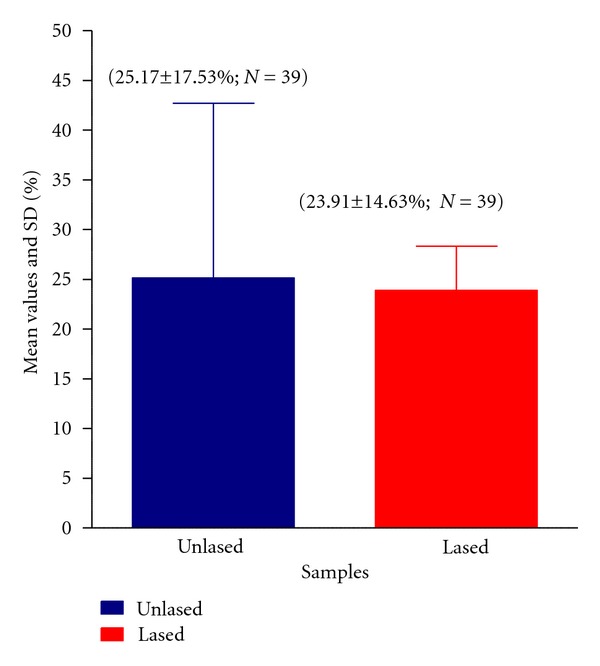
Mean values and standard deviation of the dye penetration (microleakages) in cavities filled with MTA. Lased: mean of Dye penetration in the Er:YAG lased dentine cavities (group ML); Unlased: mean of dye penetration in the unlased dentin cavities filled with MTA (group M).

The mean and standard deviation value of the percentage of dye penetration in the unlased cavities filled with MTA (group M) was 25.17 ± 17.53% (minimum value = 10; maximum value = 73). The mean value and standard deviation of the percentage of dye penetration in the group of cavities filled with MTA following Er:YAG laser irradiation (group ML) was 23.91 ± 14.63% (minimum value = 10; maximum value = 58) (Figure 2). The mean values of microleakage and dye penetration resulting from the use of an Er:YAG laser to precondition dentine were the lower of the two groups (Figure 2). All groups passed normality tests using the Kolmogorov and Smirnov method.

The difference between the percentage of dye penetration in cavities sealed with MTA (group M) that in those sealed with MTA following Er:YAG laser irradiation (group ML) was not statistically significant (paired and two-tailed *t*-test; *P* = 0.2519; *t* = 1.235 with 8 degrees of freedom; 95% confidence interval).

**Figure 3:**
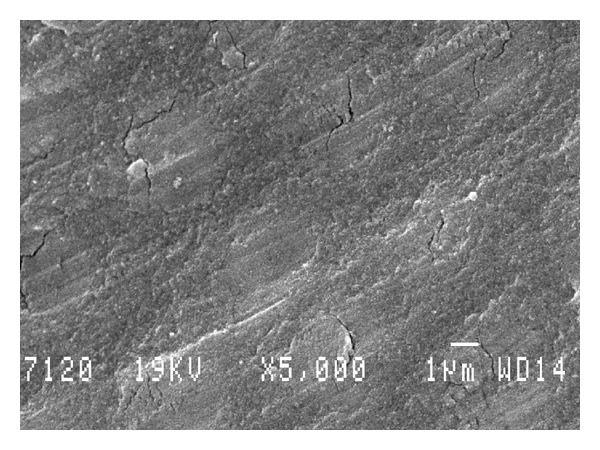
View of the dentine drilled without any complementary treatment exhibited a smear layer covering the tubules (group M). Magnification: 5000x. Scale bar = 1 *μ*m.

**Figure 4:**
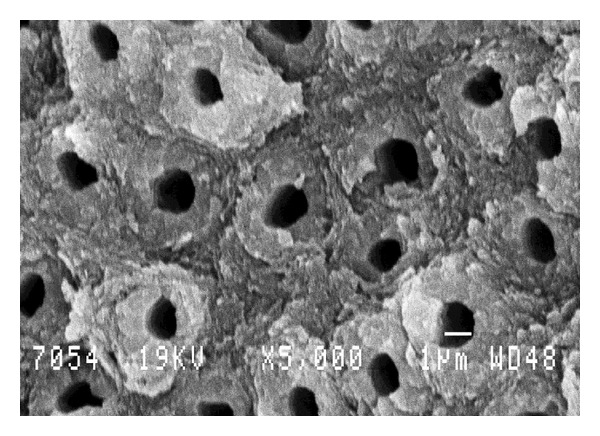
View of the dentine conditioned by Er:YAG laser (group ML), the Er:YAG laser had removed the smear layer. The tubules are totally opened. The Er:YAG laser produced a selective and preferential ablation of intertubular dentine, while the peritubular dentine (higher mineralization) was less ablated. Magnification: 5000x. Scale bar = 1 *μ*m.

The dentine drilled without any complementary treatment exhibited a smear layer covering the tubules and the total surface of the exposed dentine (Figure 3). On the other hand, on the dentine conditioned by Er:YAG laser, the Er:YAG laser had removed the smear layer (Figure 4). The tubules were totally opened. The Er:YAg laser produced a selective and preferential ablation of intertubular dentine, while the peritubular dentine (higher mineralization) was less ablated (Figure 4).

## 4. Discussion

The literature mentions several methods for the evaluation of root canal fillings. However, methylene blue is not the best choice for the measurement of the quality of sealing of root canal fillings. In our study, we evaluated the microleakage in the marginal area between the MTA and dentine. We used this dye because of its common use in several articles for the evaluation of microleakage of dentinal fillings [15–17].

Several studies showed that the use of an Er:YAG laser for cavity preparation has many advantages. Er:YAG laser (2940 nm) conditioning of perforated dentine under an air-water spray is noncontact and decontaminating [18]. The irradiation of root surfaces can remove bacterial endotoxin. Furthermore, according to our results and other studies, an Er:YAG laser beam is able to remove the dentinal smear layer and open the dentinal tubules [19, 20]. On the other hand, the conditioning of dentin by Er:YAG laser irradiation can favor cells adhesion [13]. For these reasons, we selected the Er:YAG laser for the dentinal conditioning of root perforation treatment.

The properties of MTA were studied and described. Garthner and Dorn [21] proposed the ideal characteristics of MTA: simple manipulation, radio-opacity, stability in three dimensions and in a moist environment for a long period, nonresorbability, quality adhesion to dentin, and biocompatibility with desmodontal cells.

Fournier and Bouter [9] recommended the consideration of three properties: cytotoxicity of filling material, long-term sealing strength, and simplicity of manipulation.

MTA contains a hydrophilic powder of tricalcic silicate, iron, tricalcic aluminate, tricalcic oxide, silice oxide particles, and a bismuth oxide for radio-opacity 9. Manufacturers produce two kinds of MTA (white or gray) [22]. Both offer good cellular biocompatibility and good sealing to prevent leakages [22–26].

Several studies explained that the biocompatibility of the MTA may be due to the release of calcium hydroxide in water [3, 10, 26, 27]. Other studies reported that MTA is well tolerated by periodontal tissues [28, 29]. Torabinejad et al. [3] showed the fibrous formation in contact with MTA through histological studies. Pitt Ford et al. [30] and Thomson et al. [31] observed that the MTA sealing of root perforations induces cement formation. Because of these qualities of tightness, MTA is mainly indicated for retroapical fillings [30], apexification [32], and root perforations [1, 5–7, 33]. The prognosis for a perforated root depends on the time elapsed before the sealing of the perforation, the localization and size of the perforation, and the sealing quality of the material used [34].

In accordance with the previous articles, our results demonstrate a higher sealing (in mean) of MTA to Er:YAG laser-conditioned dentine [35, 36] versus unlased MTA-sealed dentine. However, the statistical analysis did not show any significant difference. Our SEM study shows that Er:YAG laser conditioning of dentine removes the dentinal smear layer and has a less ablative effect on peritubular dentine, which has higher mineral content. The more effective sealing of MTA to Er:YAG-lased dentine may be due to the interaction between MTA components and the exposed mineral content of Er:YAG-lased dentine (chemical effects). A second explanation may be the increase of microretention in lased dentin and dentinal opened tubules (physical effect) (Figure 4).

An intimate contact between MTA and the dentine is required, but in the literature, the necessity to remove or not the smear layer prior to MTA application to exposed dentine is not clear. There is a necessity to have a standard clinical procedure mentioning the necessity or not to remove the smear layer. For this reason, we did not remove the dentinal smear layer from the exposed dentin prior to the application of MTA.

## 5. Conclusion

The use of an Er:YAG laser beam for dentinal conditioning prior to MTA filling of perforated roots or localized root crack treatments did not decrease significantly the microleakage of MTA sealing when compared to the conventional use of MTA filling.
